# An Efficient Improved Greedy Harris Hawks Optimizer and Its Application to Feature Selection

**DOI:** 10.3390/e24081065

**Published:** 2022-08-02

**Authors:** Lewang Zou, Shihua Zhou, Xiangjun Li

**Affiliations:** Key Laboratory of Advanced Design and Intelligent Computing, Ministry of Education, Dalian University, Dalian 116622, China; lixiangjun@s.dlu.edu.cn

**Keywords:** Harris Hawks Optimization, global optimization, data imbalance, feature selection

## Abstract

To overcome the lack of flexibility of Harris Hawks Optimization (HHO) in switching between exploration and exploitation, and the low efficiency of its exploitation phase, an efficient improved greedy Harris Hawks Optimizer (IGHHO) is proposed and applied to the feature selection (FS) problem. IGHHO uses a new transformation strategy that enables flexible switching between search and development, enabling it to jump out of local optima. We replace the original HHO exploitation process with improved differential perturbation and a greedy strategy to improve its global search capability. We tested it in experiments against seven algorithms using single-peaked, multi-peaked, hybrid, and composite CEC2017 benchmark functions, and IGHHO outperformed them on optimization problems with different feature functions. We propose new objective functions for the problem of data imbalance in FS and apply IGHHO to it. IGHHO outperformed comparison algorithms in terms of classification accuracy and feature subset length. The results show that IGHHO applies not only to global optimization of different feature functions but also to practical optimization problems.

## 1. Introduction

Gradient-based optimization methods have been widely applied to linear, differentiable, and continuous problems [1]. However, practical problems are increasingly nonlinear, non-differentiable, and discontinuous, rendering gradient-based methods useless. In contrast, the intelligent algorithms developed rapidly in recent years can effectively solve practical problems, although they lack rigorous mathematical derivations.

In the twenty-first century, the development of technology has also led to further research in the field of intelligent algorithms. A series of new metaheuristic algorithms have been derived. For example, Kaveh et al. [2] propose an efficient hybrid method based on the Harris Hawk Optimizer and the imperialist competitive algorithm. The Harris Hawk algorithm has an efficient exploitation strategy but performs poorly in the search for optimal solutions, which is compensated by the imperialist competitive algorithm. Song et al. [3] identified the deficiency of the global search capability of the Harris Hawk Optimizer, and they proposed the persistent-trigonometric-differences mechanism to improve the global search capability of the HHO; in addition, they improved the energy factor of the original algorithm to better balance the exploration and exploitation of the algorithm; finally, they applied it to the parameter identification problem of photovoltaic model parameter extraction. Zhong et al. [4] proposed an integrated learning Harris Hawk optimization algorithm with a terminal replacement mechanism. The authors propose to combine the comprehensive learning strategy with HHO to improve the convergence of the optimizer.

FS is an important data-preprocessing method in machine learning, but it is NP-hard [5], with a search space of $2^{n}$ for *n* features, motivating the use of approximation algorithms to obtain near-optimal solutions as well as metaheuristic algorithms [6]. Inbarani et al. [7] proposed a hybrid FS method for disease detection by combining particle swarm-based packing methods with rough set theory. Zhang et al. [8] introduced variational operators to improve the differential evolutionary algorithm and applied this algorithm to deal with FS. Tubishat et al. [9] applied SALP swarm opposite learning and local search to FS. Taradeh et al. [10] improved the GSA algorithm with a GA for more effective FS. Mafarja et al. [11] used tournament and roulette selection to enhance the search and development process of WOA. Unfortunately, most of these algorithms cannot achieve an effective balance between exploration and exploitation, which means that a better balance in terms of efficiency (search time) and outcome (feature subset length) cannot be achieved in the feature selection problem. This paper applies the improved HHO to the feature selection problem. Because the optimization mechanism of the HHO algorithm is very similar to the optimization process of the feature selection problem, each Harris Hawk can be regarded as a piece of data filled with much redundant information, and the number of data features can be regarded as the dimension of the Harris Hawk hunting space, the selection process of data features is equivalent to the optimization process of Harris Hawk hunting, and the improved algorithm can achieve a better balance between exploration and exploitation. Next, we will detail the principle of the Harris Hawk algorithm, related applications, and an overview of our proposed algorithm improvement strategy.

The Harris hawk is a raptor living in southern Arizona in the United States. Heidari et al. [12] proposed HHO in 2019 after observing cooperative feeding among Harris hawks. The algorithm has phases of exploration, transition, and development. Due to its simple principle, few hyperparameters, and fast convergence, HHO is widely used in problems involving global optimization, machine learning, medical bioinformatics, natural language processing, and image encryption. Ridha et al. [13] proposed a method to reliably and accurately identify photovoltaic model parameters by combining HHO with a flower pollination algorithm. Jia et al. [14] introduced dynamic optimization and a variational mechanism to improve HHO and applied it to the satellite image segmentation problem. Kamboj et al. [15] proposed hHHO-SCA, which combined HHO and SCA for nonconvex and nonlinear problems. Yanan et al. [16] embedded SSA in HHO to improve its search capability and applied it to FS. Chiwen et al. [17] non-linearized the escape energy factor by chaotic perturbation to balance the development and exploration of HHO. Bui et al. [18] combined HHO with artificial neural networks to optimize their performance for landslide sensitivity analysis. Roy et al. [19] applied HHO to integrated a more effectiveness and robustness model order reduction.

These applications demonstrate the wide use of HHO to solve practical problems. However, as with traditional intelligent algorithms, HHO has some drawbacks. Each phase of the search process is too homogeneous. HHO mainly relies on the escape energy of the prey, whose range is [−2, 2], to control the hunting process. When its absolute value is greater than or equal to 1, HHO performs the search phase, and when it is less than 1, HHO selects one of four exploitation strategies. This means that in its early stage, the algorithm only performs a search, and in the late stage, it only performs exploitation. This undoubtedly causes the algorithm to easily fall into premature convergence. Therefore, HHO can be improved from the aspects of the update mechanism, addition of new operations, and in combination with other algorithms. The aspect of the update mechanism is mainly reflected in exploring and developing to obtain a balance [20,21,22], which can occur through dynamic adjustment of the escape energy factor. Adding new operators can enhance performance in terms of local or global search capability [23,24]. Researchers have also tried to combine HHO with other intelligent algorithms [25,26,27].

We propose an efficient improved greedy HHO (IGHHO), starting from the update mechanism and the incorporation of new operators, replacing the development strategy of HHO with a hybrid differential and greedy strategy. A new conversion strategy makes the algorithm more flexibly switch between exploitation and search, which compares the current particle with the previous global optimum, and if it is better than the previous global optimum, the algorithm next executes the exploitation strategy; otherwise, it executes the search strategy. The performance of IGHHO was tested on the CEC2017 test set [28], which contains 29 benchmark functions. The ranking method and Wilcoxon rank-sum test results indicate significant improvement. Results on the FS problem show that the algorithm has advantages in practical applications. The major involvement of this study is explained as below:

- A novel efficient IGHHO for global optimization and feature selection.
- The proposed IGHHO has efficient flexibility to switch between search and development and has strong development capabilities.
- The performance of IGHHO was better than with other state-of-the-art optimization techniques.
- The IGHHO is applied for feature selection, and we verify the performance of this algorithm in an open dataset.

The remainder of this article is organized as follows. Section 2 describes the principle of HHO and its model, and Section 3 discusses the details of IGHHO and its motivation. Section 4 describes experiments to test the function. The method is applied to FS in Section 5. Section 6 relates our conclusions and proposes future research directions.

## 2. An Overview of HHO

HHO is mainly inspired by the cooperative behavior and the way of chasing during the Harris Hawk raid [12].

### 2.1. Exploration

At the beginning of a hunt, Harris hawks randomly stay on high ground to find and track prey by eye. HHO models this process as Harris hawks randomly perching through two equal-opportunity strategies,

(1) $$X\left(t+1\right)=\left\{\begin{array}{c} X_{rand}\left(t\right)-w_{1}\left|X_{rand}\left(t\right)-2w_{2}X\left(t\right)\right|,\;random\geq 0.5\\ \left(X_{rab}\left(t\right)-X_{m}\left(t\right)\right)-w_{3}\left(L+w_{4}\left(U-L\right)\right),\;random<0.5 \end{array}\right.$$

where X(t+1) is a Harris hawk’s position in the next iteration; $X_{rab}\left(t\right)$ is the position of the prey (i.e., the individual with the optimal fitness value); X(t) is a Harris hawk’s position in the current iteration; $w_{1}$, $w_{2}$, $w_{3}$, $w_{4}$, and random are random numbers in the range (0,1); *U* and *L* are the upper and lower bound, respectively, of the variables indicating the activity of the Harris hawk population; $X_{rand}\left(t\right)$ is a randomly selected position in the population; and $X_{m}\left(t\right)$ is the individual’s average position, calculated from Equation (2), where Number is the number of Harris hawks in the population.

(2) $$X_{m}\left(t\right)=\frac{1}{Number}\sum_{k=1}^{number}X_{k}\left(t\right)$$

### 2.2. Exploration to Exploitation

HHO switches from exploration to exploitation according to the change in the escape energy of the rabbit, based on which it chooses exploitation strategies,

(3) $$Esc=2Esc_{0}\left(1-\frac{t}{T_{max}}\right)$$

where Esc is the escape energy of the rabbit, with initial value $Esc_{0}$, which varies randomly within (−1, 1); $T_{max}$ is the maximum iterations; and *t* is the current iteration number.

### 2.3. Exploitation

HHO uses the following strategies to simulate the exploitation process.

#### 2.3.1. Soft and Hard Encircle

When the prey has no chance to escape (i.e., when the random number *r* (control factors in Section 2.4) is greater than 0.5), the flock selects Equation (4) to round up the prey if the absolute value of its escape energy is greater than or equal to 0.5, and Equation (5) otherwise:

(4) $$X\left(t+1\right)=\Delta X\left(t\right)-Esc|JumpX_{rab}\left(t\right)-X\left(t\right)|$$

(5) $$X\left(t+1\right)=X_{rab}\left(t\right)-Esc\left|\Delta X\left(t\right)\right|$$

where ΔX(t) is the difference between the optimal individual and current individual, which is calculated by Equation (6), and Jump is the distance of random jumps during prey escape, which is calculated by Equation (7), where $w_{5}$ is a random number within (0, 1).

(6) $$\Delta X\left(t\right)=X_{rab}\left(t\right)-X\left(t\right)$$

(7) $$Jump=2(1-w_{5})$$

#### 2.3.2. Soft Encircle with Advanced Fast Dives

When the prey is about to escape (random is less than 0.5) and the absolute value of escape energy is greater than 0.5, HHO uses a greedy approach to simulate a Harris hawk flock surrounding the prey through Equation (10).

(8) $$P=X_{rab}\left(t\right)-Esc\left|JumpX_{rab}\left(t\right)-X\left(t\right)\right|$$

(9) Q=P+S∗LF(D)

(10) $$X\left(t+1\right)=\left\{\begin{array}{cc} P, & \;\mathrm{if}\;f(P)<f(X(t))\\ Q, & \;\mathrm{if}\;f(Q)<f(X(t)) \end{array}\right.$$

Equations (8) and (9) obtain the alternative positions of the particle, and *P* and *Q* are represented by the alternative positions of the particle, respectively. Judging by Equation (10), if f(P) is smaller than f(X(t)), *P* is selected as the official position of the particle, and if f(Q) is smaller than f(X(t)), *Q* is selected as the official position of the particle. If both are less than f(X(t)), *P* is selected as the official position of the particle according to the order of program execution. If neither is smaller than f(X(t)), the particle position is not updated.

#### 2.3.3. Hard Encircle with Advanced Fast Dives

When the prey is about to escape (random is less than 0.5) and the absolute value of its escape energy is less than or equal to 0.5, HHO uses a greedy approach to simulate the flock pouncing on the prey through Equation (13).

(11) $$P=X_{rab}\left(t\right)-Esc\left|JumpX_{rab}\left(t\right)-X_{m}\left(t\right)\right|$$

(12) Q=P+S∗LF(D)

(13) $$X\left(t+1\right)=\left\{\begin{array}{cc} P, & \;\mathrm{if}\;f(P)<f(X(t))\\ Q, & \;\mathrm{if}\;f(Q)<f(X(t)) \end{array}\right.$$

### 2.4. The Overall of HHO

**Figure 1:**
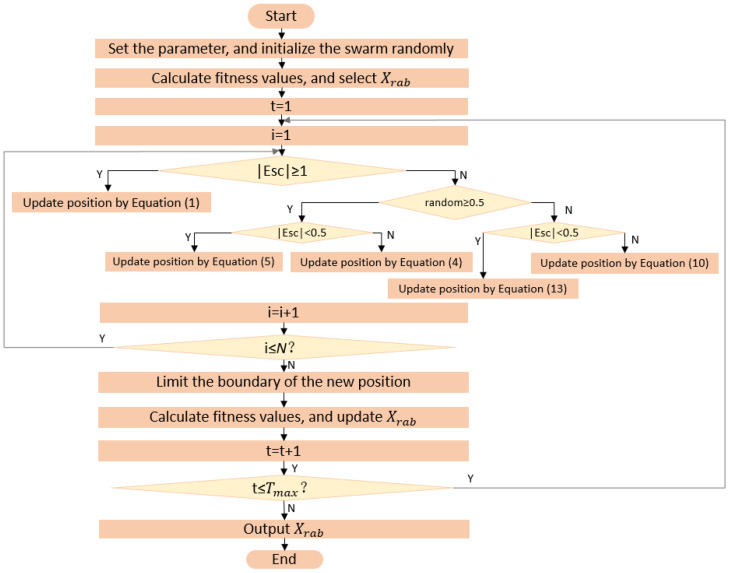
Flowchart of HHO.

Figure 1 shows the flowchart of the HHO algorithm. HHO relies heavily on the search and development of escape energy E control algorithm. Exploration is performed when |Esc|≥1, and exploitation is performed otherwise.

## 3. Proposed Method

### 3.1. Differential Perturbation Strategy

The DE algorithm is known for its good population diversity [29], where each iteration of its particles is perturbed by the weighted difference of two randomly selected particles. This makes the algorithm rich in population diversity. We propose Equation (14) for the differential perturbation of particles,

(14) $$X_{i}^{d}\left(t+1\right)={pbest}_{i}^{d}\left(t\right)+2*rand*\left(gbest^{d}\left(t\right)-{pbest}_{i}^{d}\left(t\right)\right)$$

where *d* is the dimension of the particle, an integer in the range [1,Dim], where Dim is the total dimension of the search space; rand is a uniformly distributed random number in the interval (0, 1); and *i* is the index number of the current particle.

The first part of Equation (14) is the historical best position of the *i*th particle, and the second part is the weighted difference between its best position in the whole population and its historical best position. When rand is large, the particle convergence speed is high, and when rand is small, the particle will perform local search based on its historical best position. Updating the particles by Equation (14) can direct them toward the region where the global optimal solution is more likely to be found based on their historical optimal positions. This enhances the algorithm’s optimal search ability while enriching the population diversity.

Our proposed differential perturbation strategy has several advantages: (1) Instead of using randomly selected particles, it makes full use of the ‘cognition’ of the particles (historical optimum) and the ‘social cognition’ of the population. (2) The method takes a random number from (0, 2) as the weight, which effectively balances exploration and exploitation to avoid convergence that is too fast and that falls into a local optimum, and exploration does not waste the ‘social cognition’ of the whole population.

### 3.2. Greedy Strategy

To fully exploit the properties of inter-particle collaboration and knowledge sharing, we propose a development strategy different from differential perturbation,

(15) $$P=gbest-E*(gbest-X_{i})$$

(16) $$temp_{1}=gbest-\alpha_{1}*\left|gbest-X_{i}\right|$$

(17) $$temp_{2}=mean\_best_{i}-\alpha_{2}*\left|mean\_best_{i}-X_{i}\right|$$

(18) $$Q=\frac{temp_{1}+temp_{2}}{2}$$

where *E* in Equation (15) is the escape energy of the particle; $\alpha_{1}$ and $\alpha_{2}$ in Equations (16) and (17) are both weight factors, which can be calculated from Equation (20); $mean\_best_{i}$ in Equation (17) can be calculated from Equation (21), which serves to extract the *k* particles with better fitness values than the current particle from the list of individual historical best of the population.

(19) $$a=2*(1-\frac{t}{T})$$

(20) α=a∗(2∗rand−1)

(21) $$mean\_best_{i}=\sum_{m=1}^{k}pbest_{k}$$

It is worth noting that the operations in Equations (15)–(21) are performed for each dimension of a particle. In Equation (15), the escape energy E decreases linearly with the number of iterations, implying that the particle converges increasingly quickly. Equations (16)–(18) are inspired by the GWO, using multiple better particles to guide the direction of the remaining particles in their search for superiority. However, GWO uses the three best particles, which will inevitably lead to premature convergence [30], and there are more parameters and complexity in GWO compared to IGHHO. In general, each particle can provide valuable information, which is called the particle’s own “advantage” [31]. For example, particles with good fitness can indicate that the region in which they are located is likely to have the global optimum, while a particle with a poor fitness can indicate that the current region has a low probability of having a global optimum. To improve the global search efficiency, we should avoid being near these regions. To take full advantage of these “advantages”, we use the optimal particle in the population and the mean of k particles with better fitness than the current particle as learning objects [32]. This reflects the idea of cooperation and knowledge sharing of evolutionary algorithms to a certain extent. Therefore, our proposed equations have the following characteristics: (1) fewer and simpler parameters compared to GWO; so their impact is slightly reduced; (2) we borrow the greedy strategy of the GWO algorithm, drawing on the optimal particles in the population and the “advantage” of other particles to enhance the algorithm’s optimality-seeking stability.

Therefore, in this strategy, the particles are updated according to Equation (22),

(22) $$X\left(t+1\right)=\left\{\begin{array}{cc} P, & f(P)<f(Q)\\ Q, & f(P)>f(Q) \end{array}\right.$$

### 3.3. Hybrid Differential Perturbation with Greed

IGHHO exploits the region where the particles are located by Equation (14) or Equation (22) under the condition that the particles adapt better than before. While Equation (22) has a stronger exploitation ability, Equation (14) has a stronger search capability. If Formula (22) is used extensively in the early stage to update the particle position, the particle will fall into local optimum prematurely. If Formula (14) is used extensively to update the particle position in the later period, particle optimization will be too slow, resulting in low efficiency. To balance them, we use the fluctuation of the sine function [33] to alternately update particle positions using Equations (14) and (22), as shown in Algorithm 1.

where $C_{r}$ is calculated as,

(23) $$C_{r}=0.5*\left(\sin\left(2\pi *0.25*t+\pi \right)*\frac{t}{T}+1\right)$$

**Algorithm 1** Updating way of exploitation.
1:Calculate $C_{r}$ according to Equation (23)2:**if** rand < $C_{r}$ **then**3:    update particle position by Equation (14)4:
**else**
5:    update particle position by Equation (22)6:
**end if**



**Figure 2:**
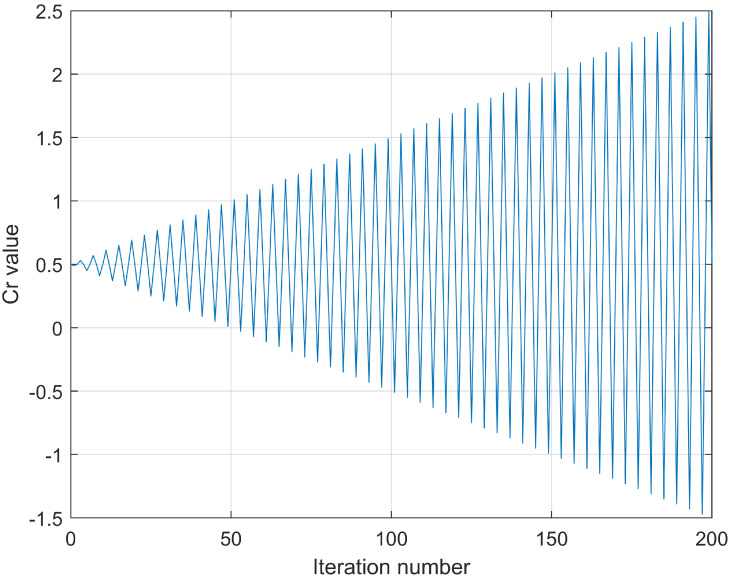
Distribution graph of $C_{r}$.

As Figure 2 shows, the $C_{r}$ value is the function of the number of iterations *t* distribution; as shown in Figure 2, the value of $C_{r}$ fluctuates around 0.5, and the fluctuation range of $C_{r}$ is gradually increasing with iteration number. This balances the number of executions of the two strategies so that the particles do not converge too quickly in the early and strongly converge in the latter.

### 3.4. Conversion Exploration and Exploitation

The transition between development and search in HHO relies only on the determination of the escape energy factor, which varies linearly during iteration, making the algorithm unable to switch flexibly between development and search. Aydilek [34] proposed a hybrid strategy to combine FA and PSO based on the current particle’s superiority or inferiority. The current particle is compared with the previous global optimum and updated using FA if it is better; otherwise, it is updated using PSO. However, doing so would result in a larger proportion of runs for one strategy than for the other and not taking full advantage of the other strategy. Our proposed algorithm performs the development phase if the current particle is better than the previous optimal value; otherwise, it performs the search phase. This allows the particle to take full advantage of previous information and balances development and search.

According to the above description, the development and search conversion strategy proposed in this paper is as follows:

(24) $$Conversion\;Strategy=\left\{\begin{array}{c} Exploration,f\left(X_{i}\right)<f\left(pbest_{i}^{t-\gamma }\right)\;or\;t\leq 5\\ Exploitation,otherwise \end{array}\right.$$

where $f(pbest_{i}^{t-\gamma })$ is the optimal value that could be achieved in the previous γ iterations of the *i*th particle, and *t* is the current number of iterations.

### 3.5. Overall Algorithm

**Figure 3:**
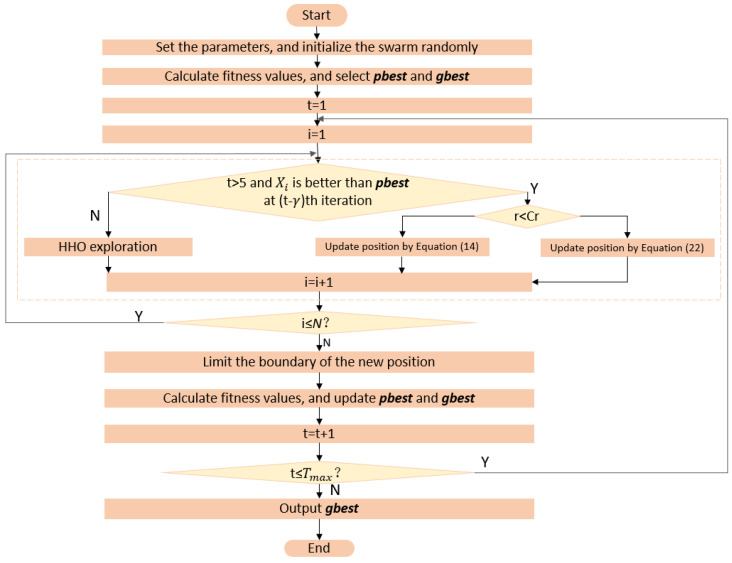
Flowchart of IGHHO.

Figure 3 shows the flowchart of IGHHO, which has four input parameters: number of particles, maximum number of iterations, search space boundary value, and problem dimension. The IGHHO proposed in this paper differs from the HHO mainly in that the HHO focuses more on search in the early stage guided by escape energy and more on development in the later stage, making the algorithm easy to fall into premature convergence and poor performance; the IGHHO is flexible to switch between the two, switching strategies if the particles do not achieve better performance after γ iterations. In the exploitation stage, the introduction of two exploitation methods makes the algorithm more flexible based on strengthening the exploitation ability and enriching the population diversity. Two methods in the development stage add flexibility by strengthening development ability and enriching population diversity.

### 3.6. Computational Complexity Analysis of the Algorithm

In general, the time complexity of the metaheuristic algorithm is mainly composed of three parts as follows:

1. The initialization of the population. The time complexity of this part is mainly determined by the population size N and the population dimension D, which generally does not exceed O (N×D).
2. The computation of the fitness of the initial population. The time complexity of this part is mainly determined by the population size N and the target cost generated by the problem, which generally does not exceed O (N×Cost).
3. Main loop. The time complexity of this part is mainly determined by the number of iterations T, the population size N, the population dimension D, and the target cost generated by the problem, which generally does not exceed O (T×N×D+T×N×Cost).

Moreover, the time complexity of our algorithm also consists of these three main components:

1. Population initialization. The time complexity of this part is comparable to that of other algorithms, O (N×D).
2. Initial population fitness calculation. The time complexity of this part is also comparable to other algorithms, O (N×Cost).
3. In the main loop. As can be seen in Figure 3, the time complexity of this part of the algorithm mainly consists of particle position update and fitness calculation. The particle position is updated by the search strategy and the development strategy alternately. When the algorithm does not satisfy the judgment condition, the left branch is executed; that is, the search strategy is executed according to the original Harris Hawk algorithm. When the algorithm satisfies the judgment condition, the right branch is executed, that is, the development strategy is executed by Equation (14) or Equation (20). The time complexity of this part does not exceed O (T×N×D), and the adaptation calculation does not exceed O (T×N×Cost). Therefore, the time complexity of the main loop can be expressed as O (T×N×D+T×N×Cost), which is also comparable to other algorithms.

We can conclude from the above analysis that the time complexity of the proposed IGHHO is comparable to other algorithms.

The computational complexity of the algorithm consists of three main parts: initializing the population position, updating the population position, and calculating the particle fitness. Our proposed IGHHO algorithm has roughly the same framework as the HHO algorithm, so the computational complexity in these parts is the same. It follows that O (IGHHO) = O (Initialization Harris hawks) + O(Estimate the fitness of hawks) + T*O (Update the position of all hawks). Where O (Harris hawks initialization step) = O (N), and O(Estimate the fitness of hawks) = T*O (N), O (Update the position of all hawks) = O (N*D). So, the total time complexity is O (IGHHO) = O (N) + T*O (N) + T*O (N*D) = O (T*N*D).

## 4. Experimental Results and Analysis

### 4.1. Experimental Design and Parameter Settings

To verify the global search capability of the IGHHO, we tested it on the CEC-2017 test set [28], which contains 29 test functions, of which f1–f2 are single-peaked, f3–f9 are multi-peaked, f10–f19 are hybrid, and f20–f29 are composite.

All experiments were compiled and run in MATLAB R2020b on a Windows 10 platform using a Core i7-6700HQ CPU 2.60 GHz with 16 GB of RAM.

We compared IGHHO with SCADE [35], IWOA [36], BMWOA [37], CDLOBA [38], RCBA [39], BLPSO [40], and CLPSO [41]. We set the number of population particles for each algorithm to 30, the particle dimension to 30, the boundary values of each dimension to [−100, 100], and the maximum iterations to 1000. We ran each algorithm 30 times and took the average as the final result. Table 1 shows the parameter settings for all algorithms.

### 4.2. Experimental Results and Analysis

Table 2, Table 3 and Table 4 list the experimental results of IGHHO and comparison algorithms in the same environment. Among them, the data of comparison algorithm are quoted from the simulation results of Zhangze et al. [42]. From Table 2, we can see that IGHHO ranks first on both single-peaked functions, in the top three on both multi-peaked functions, and first on f4 and f7. This shows that IGHHO has good results on the global optimization search problem, and it has a strong ability to jump out of local optima.

In Table 3, IGHHO ranks first on most hybrid functions, produces order-of-magnitude differences from second place on f12, f14, and f18, and is not far from first on poorly performing functions f16 and f19. This demonstrates the effectiveness of our strategy.

From Table 4, it can be seen that the results of IGHHO rank first on eight of the nine composite functions, and IGHHO is tied with the comparison algorithm in terms of variance.

Overall, IGHHO is superior to BMWOA, SCADE, CDLOBA, CLPSO, IWOA, BLPSO, and RCBA on all types of test functions in CEC-2017. This is also evident from the results of the Wilcoxon signed-rank test [43] in Table 5, where *n*/*w*/*t*/*l* indicate the number of functions on which IGHHO is superior, equal, or inferior, respectively, to the comparison algorithm in *n* problems. It can be seen that IGHHO is superior to all comparison algorithms.

### 4.3. Convergence Analysis

**Figure 4:**
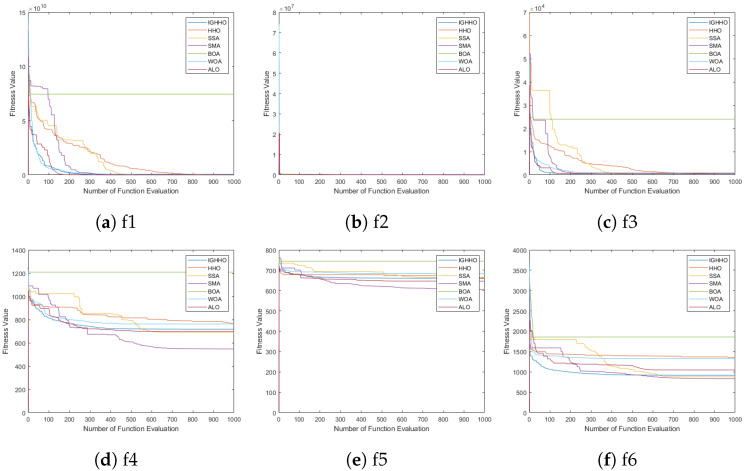

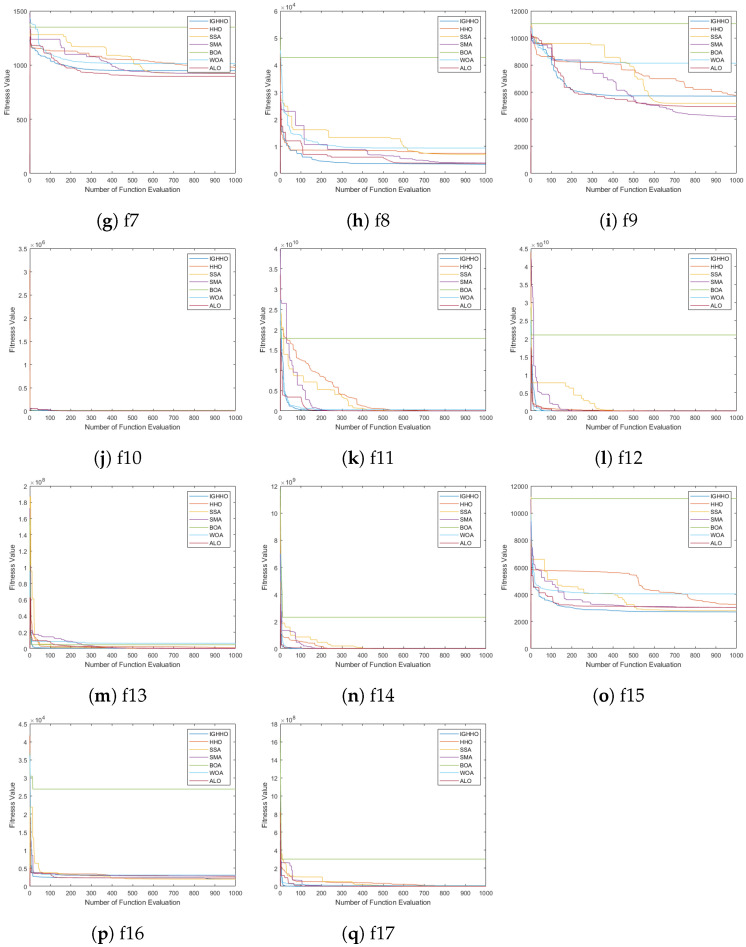
Comparison of convergence curves of IGHHO and related methods (1).

**Figure 5:**
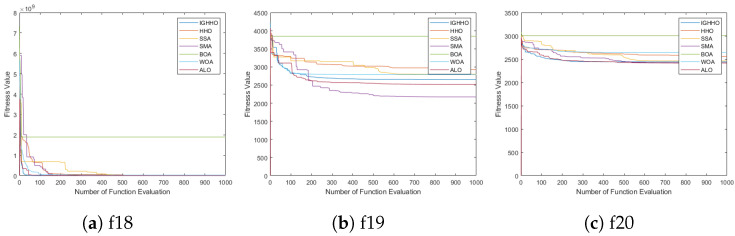

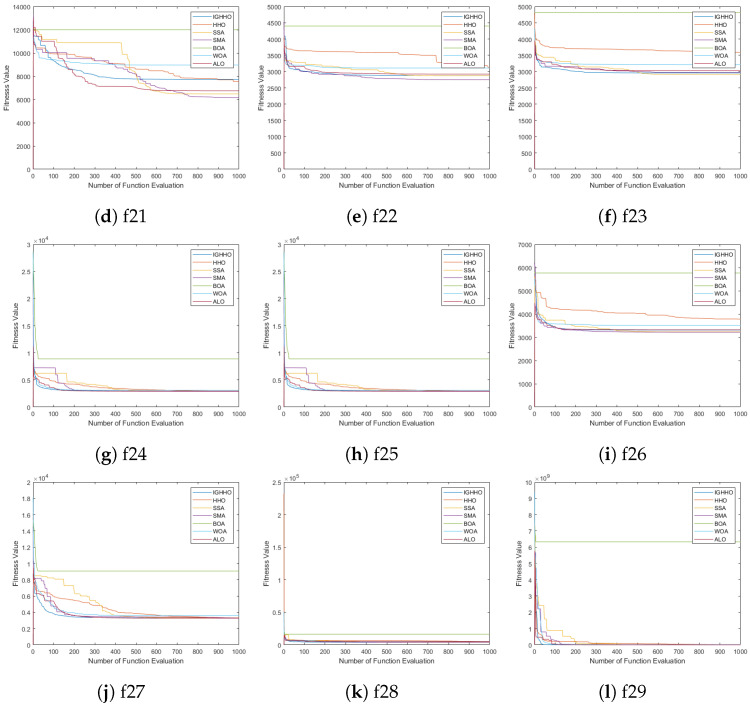
Comparison of convergence curves of IGHHO and related methods (2).

In order to better show the optimization performance of IGHHO, this section sets up experiments to analyze the convergence of the proposed algorithms. We compare the proposed algorithm with the recently proposed HHO [12], SSA [6], SMA [44], BOA [45], WOA [11], and ALO [46] for analysis. For fairness, the population size of each algorithm is set to 30, the maximum number of iterations is set to 1000, the particle dimension is set to 30, the search space is [−100, 100], and the evaluation function is cec2017. The experimental results are shown in Figure 4 and Figure 5 shown, and it is obvious that the proposed IGHHO has better convergence in the optimization process. For example, IGHHO ranks in the top 2 among the comparative algorithms in terms of convergence speed of functions f1, f2, f3, f4, f6, f7, f8, f9, f10, f11, f12, f13, f14, f15, f16, f17, f18, f20, f21, f22, f23, f24, f25, f27, f28, and f29. The above indicates that the proposed algorithm convergence is very competitive among the comparative algorithms.

## 5. Application to FS

FS is an integral part to improve classification performance by removing irrelevant and redundant features for fast computation [47]. The wrapper method based on the population intelligence algorithm is widely used due to its simple algorithm and ease of implementation. The method treats the model as a black box [48], evaluates the feature subset using classifiers or other learning models, and continuously improves its quality. Based on this, we apply IGHHO to the FS problem.

### 5.1. Model Description

We use the feature subset obtained by evaluating the K-Nearest Neighbor (KNN) classifier. Considering the impact of the data imbalance problem on feature selection [49,50], we designed the objective function by weighting the second-order classification error rate and the length of the feature subset,

(25) $$fitness=\mu \cdot balanced\_error+\left(1-\mu \right)\cdot \frac{sf}{nf}$$

where sf is the length of the selected feature subset; nf is the total number of features in the dataset; μ is a factor to balance the classification error rate with the length of the feature subset, and

(26) $$balanced\_error=\frac{1}{n}*\sum_{k=1}^{n}{\left(1-\frac{TP_{k}}{\left|S_{k}\right|}\right)}^{2}$$

where *n* is the number of problem classes, $TP_{k}$ is the number of correctly classified instances in class *k*, and $S_{k}$ is the number of all instances in class *k*. To better classify classes with few instances, we use the square of the classification error rate to penalize poorly performing classes.

The reason for this consideration is that some classes in the dataset have very few instances, while others have very many instances. For example, for a binary classification problem with 10 instances, problem A has only one instance, while problem B has nine instances. The classifier can easily achieve 90% classification accuracy by simply classifying all instances as problem B, which seems efficient, but the algorithm will perform poorly on real-world problems. To consider only the classification error rate will cause the selected feature subset to contain more redundant features, which will greatly increase the algorithm’s computational complexity, especially for high-dimensional problems. Therefore, we consider the size of the feature subset as an objective function so as to minimize the ratio of the number of selected features to that of all features.

### 5.2. Dataset Descriptions

To verify the performance of IGHHO in FS, we tested it on 13 publicly available datasets in the UCI Machine Learning Repository [51]. As shown in Table 6, these datasets are low-, medium-, and high-dimensional, and there are fewer samples of high-dimensional data, which are highly unbalanced datasets, which are more problematic in FS.

### 5.3. Experimental Configuration and Parameter Settings

Since some data have fewer samples, we used five-fold cross-validation, dividing the dataset into five parts, taking four for training and one for testing. Only the training set was used for FS, and the test set was input to the KNN model to evaluate the FS performance.

We compared our FS method to HHO [12], EPO [52], SSA [6], SMA [44], BOA [45], WOA [11], and ALO [46], which were based on advanced metaheuristics. For a fair comparison, the algorithms had consistent configurations, with a population size of 10, maximum of 50 iterations, 20 runs to take the mean value, and KNN parameter set to 5.

### 5.4. Experimental Results and Analysis

Experimental results comparing IGHHO with other algorithms on the FS problem are presented in Table 7. From the total classification accuracy results, the proposed IGHHO algorithm ranks in the top three on all datasets, ranks first on the datasets Zoo, Waveform_noise, Lung, Sonar, Isolet, Leukemia, Arcene, and Colon, and even achieves 100% classification accuracy on the dataset Leukemia. In contrast, the EPO algorithm ranked first on only three datasets, and its classification accuracy was only 0.57% better than IGHHO; the SMA algorithm ranked first on only two datasets, Clean1 and Colon, and it is worth noting that on the dataset Clean1, IGHHO achieved only 0.1% less classification accuracy than the first place, while on the dataset Colon, IGHHO tied with SMA for first place; the ALO algorithm ranked first only on dataset CNAE, and again, IGHHO was only 1.57% less than it; HHO, SSA, BOA, and WOA algorithms did not achieve first place on any dataset. In terms of the average ranking, our proposed improved algorithm ranks 1.58 on average, pulling away from the second place of 2.88 and the third place of 3.69. This all indicates that our improved algorithm is dominant relative to the comparison algorithm.

**Figure 6:**
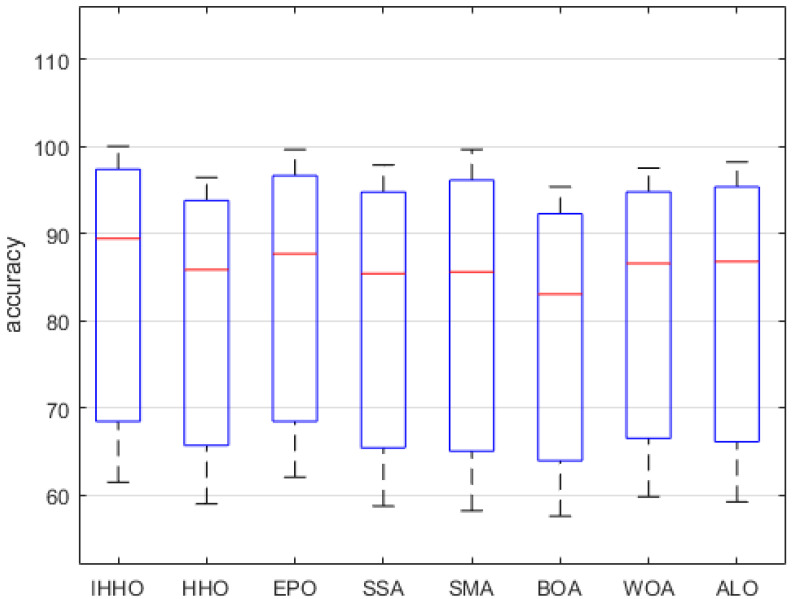
Boxplots of IGHHO versus other optimization methods for classification accuracy.

On the other hand, Figure 6 gives a box plot analysis of the classification accuracy between IGHHO and the comparison algorithm. We can see that the average classification accuracy of IGHHO is nearly 90%, which is much higher than that of the comparison algorithm. The optimal and lowest classification accuracy are also dominant compared with the comparison algorithm. Combined with the above analysis, the IGHHO algorithm proposed in this paper has some advantages over the traditional optimization algorithm in improving the classification accuracy of feature selection.

**Figure 7:**
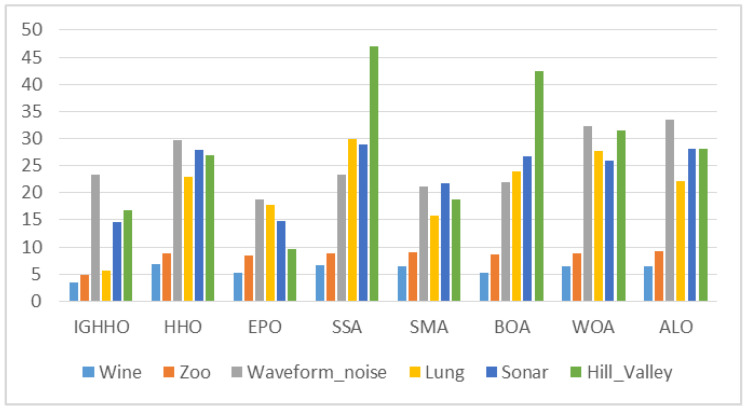
Comparison of selected feature length of IGHHO and related methods (With dataset Wine, Zoo, Waveform_noise, Lung, Sonar and Hill_Valley).

**Figure 8:**
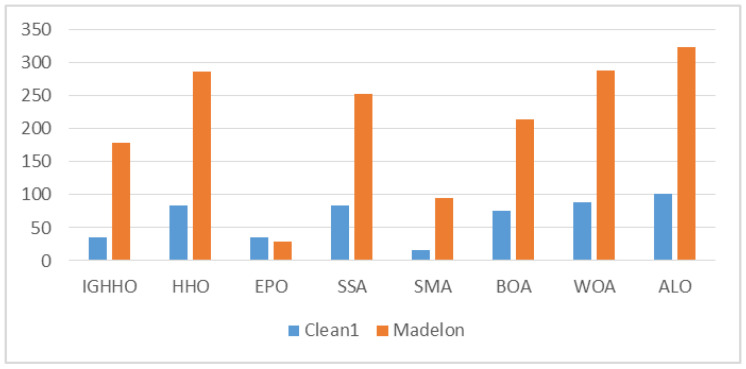
Comparison of selected feature length of IGHHO and related methods (With dataset Clean1 and Madelon).

**Figure 9:**
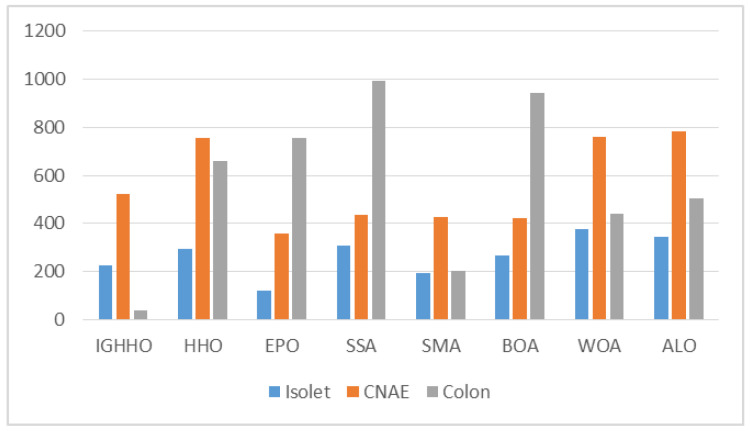
Comparison of selected feature length of IGHHO and related methods (With dataset isolet, CNAE and Colon).

**Figure 10:**
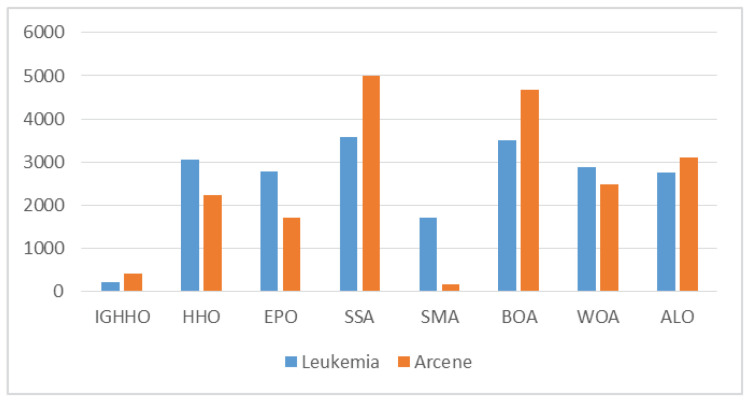
Comparison of selected feature length of IGHHO and related methods (With dataset Leukemia and Arcene).

From the average size of the features selected by IGHHO and the comparison algorithms, as shown in Figure 7, Figure 8, Figure 9 and Figure 10, it can be observed that IGHHO achieves the shortest feature subset length on six datasets, EPO on five, and SMA on two. However, looking at its data, the IGHHO algorithm achieved a feature subset length nearly 32% shorter than the second place on the dataset Wine, and the final filtered feature subset length was 43% shorter overall compared to other comparative algorithms; nearly 43% shorter than the second place on Zoo and 45% shorter overall; nearly 65% shorter than the second place on Lung and 75% shorter overall; nearly 82% shorter than the second place on Colon and 94% shorter overall; nearly 88% shorter than the second place on Leukemia and 92% shorter overall; nearly 82% shorter than second place on Colon and 94% shorter than overall; and nearly 88% shorter than second place on Leukemia and 92% shorter than overall. For the dataset Waveform_nosie, IGHHO did not achieve outstanding feature subset lengths; for the dataset Sonar, IGHHO achieved about the same results as EPO, while both had a significant advantage over the other comparison algorithms (nearly 45% reduction); for the dataset Hill Valley, IGHHO achieved second place; for the dataset Clean1, IGHHO achieves the second place with an average reduction of 61% compared to the 4th, 5th, 6th, 7th, and 8th places; for the dataset Madelon, IGHHO ranks third and has a big disadvantage compared to the first place, but it still achieves good results compared to the other comparison algorithms (34% reduction in feature subset length). Finally, for Arcene, a dataset with a very large number of features, IGHHO ranks second and has a significant advantage (almost 86% reduction). Overall, the IGHHO algorithm proposed in this paper has some advantages over the comparison algorithm in terms of the length of the selected feature subset.

Table 8 compares the average computation time of IGHHO and other algorithms on the FS problem, from which it is clear that SMA is the fastest, and IGHHO shows a slight overall advantage. In terms of the IGHHO algorithm and the HHO algorithm, IGHHO is almost twice as faster as HHO on the medium-dimensional datasets Clean1, Madelon, Isolet, and CNAE and ranks in the top three, which is a competitive advantage over other comparative algorithms; on the other hand, it is about three times faster than the HHO algorithm on the high-dimensional datasets Colon, Leukemia and Arcene and ranks first (second on the dataset Colon), which is a significant advantage over other comparative algorithms. IGHHO’s good performance in operational efficiency is not surprising because we introduced a differential perturbation and greedy strategy in the development stage of the algorithm, which gives the algorithm the possibility to explore unknown regions while having high-intensity development capability in the late iteration. This dramatically accelerates the operational efficiency of the algorithm. This advantage is highly prominent when dealing with high-dimensional problems. In summary, the improvement of the HHO algorithm is successful.

## 6. Discussion

The purpose of this study is to propose an efficient search mechanism to solve the feature selection problem for low and high-dimensional datasets. Using a hybrid approach, this study proposes integrating greedy and differential in the development phase of HHO and introducing a dynamic conversion strategy in the conversion mechanism of algorithm development and search to enhance the global search capability of the algorithm while giving it a non-weak local search capability. Through the previous experimental analysis and comparative study, with the help of numerical optimization and feature selection problems, we demonstrate the effectiveness of the proposed method.

Our proposed method has the following advantages:

- IGHHO can efficiently search for optimization problems of varying difficulty and complexity. The optimization solutions generated by IGHHO have better fitness values compared to various other advanced optimization methods, as shown in Table 2, Table 3 and Table 4 and Table 7.
- Statistically, the solutions generated by IGHHO are significantly different from those generated by other advanced optimization methods, as shown in Table 5.
- Although there is no difference between IGHHO and HHO in terms of computational complexity, IGHHO can produce more efficient solutions than HHO, especially for high-dimensional problems; see Table 8.
- To verify the effectiveness of IGHHO for the feature selection problem, the datasets selected for this study vary widely in feature size, from 13 features to 10,000 features, providing an adequate test environment for validating the optimization strategy; see Table 6.
- In terms of the length of the filtered feature subsets, IGHHO achieved good results on all datasets, with an overall minimum average reduction of 34% and a maximum of 94% compared to other comparative methods. See Figure 7, Figure 8, Figure 9 and Figure 10.
- In terms of classification accuracy, IGHHO filtered feature subsets helped the learning algorithm KNN produce an average accuracy of 89.42% on all classification datasets, with a maximum accuracy of 100%; see Table 7 and Figure 6.
- The design principle of IGHHO is so simple that researchers can easily build on our algorithm with further enhancements.

In addition to the advantages, our proposed IGHHO has the following limitations:

- IGHHO is derived from HHO, and thus, it is relatively computationally expensive compared to other optimization methods for low-dimensional problems; see Table 8.
- IGHHO is a stochastic-based optimization technique, and the subset of features it filters out may vary from run to run, which inevitably confuses users.
- In this study, the packing-based KNN algorithm is used as the learning method for feature selection, but the KNN algorithm has unavoidable limitations such as slow running efficiency.

## 7. Conclusions and Future Directions

We proposed an IGHHO. We improved the development phase using differential perturbations and a greedy strategy to enhance population diversity. A new transformation strategy made the algorithm flexible in switching between search and development, which enhanced its global search capability. The performance of IGHHO was verified on the CEC2017 test set with different features. In addition, we proposed a new objective function to address data imbalance in FS and applied IGHHO to the FS problem to verify its effectiveness in practical applications. The obtained results demonstrated the improvement over the HHO algorithm in computational accuracy and search efficiency. The proposed algorithm was seen to be efficient and reliable for practical optimization problems. However, IGHHO has some drawbacks. In the FS problem, although IGHHO generally outperformed comparison algorithms, it did not account for the majority of first rankings. In the next work, we will try to study a more efficient optimization strategy, try to propose a new improve algorithm with better performance, and apply it to other practical problems.

## Figures and Tables

**Table 1:** Algorithm parameter settings.

Method	Population (10${}^{1}$)	Maximum Generation (10${}^{2}$)	Other Parameters
IGHHO	3	10	ub = 100; lb = −100
SCADE	3	10	$C_{min}$ = 0.2; $C_{max}$ = 0.8; CR = 0.8
IWOA	3	10	$w_{1}$ = [2 0]; $w_{2}$ = [−2 −1]; b = 1; $m_{u}$ = 0.1
BMWOA	3	10	$w_{1}$ = [2 0]; $w_{2}$ = [−2 −1]; b = 1
CDLOBA	3	10	$Q_{min}$ = 0; $Q_{max}$ = 2
RCBA	3	10	$Q_{min}$ = 0; $Q_{max}$ = 2
BLPSO	3	10	$C_{1}$ = [0.2 0.9]; $C_{2}$ = 1.496; $C_{3}$ = 1; Esc = 1
CLPSO	3	10	$C_{1}$ = [0.2 0.9]; $C_{2}$ = 1.496

**Table 2:** Comparison results with variants of traditional optimization algorithms on unimodal functions and simple multimodal functions.

Func		IGHHO	SCADE	CLPSO	BLPSO	IWOA	BMWOA	CDLOBA	RCBA
f1	Avg (10${}^{3}$)	4.68	26,000,000	1,510,000	3,280,000	896,000	273,000	12.2	285
	Std (10${}^{3}$)	4.96	3,490,000	334,000	597,000	646,000	118,000	5.18	93.8
	Rank	1	8	6	7	5	4	2	3
f2	Avg (10${}^{4}$)	1.53	7.25	12.8	9.63	21.4	6.83	3.23	4.07
	Std (10${}^{3}$)	4.22	5.74	27.8	21.4	646,000	7.50	15.2	23
	Rank	1	5	7	6	8	4	2	3
f3	Avg (10${}^{2}$)	5.06	53.5	9.50	9.07	6.79	6.15	5.10	4.98
	Std (10${}^{1}$)	2.74	135	10.4	7.48	6.49	4.48	3.22	3.18
	Rank	2	8	7	6	5	4	3	1
f4	Avg (10${}^{2}$)	7.28	8.60	7.36	7.53	7.96	8.00	8.67	7.98
	Std (10${}^{1}$)	4.36	1.79	2.22	1.47	5.57	3.97	7.60	6.64
	Rank	1	7	2	3	4	6	8	5
f5	Avg (10${}^{2}$)	6.55	6.69	6.29	6.30	6.68	6.65	6.69	6.75
	Std	8.15	8.22	4.19	3.35	12.8	7.57	8.79	10.5
	Rank	3	6.5	1	2	5	4	6.5	8
f6	Avg (10${}^{3}$)	1.04	1.24	1.03	1.09	1.25	1.22	2.61	1.92
	Std (10${}^{1}$)	8.27	2.80	2.30	2.54	8.83	10.5	30.3	32.7
	Rank	2	5	1	3	6	4	8	7
f7	Avg (10${}^{2}$)	9.69	11	10.4	10.5	10.4	10.1	11.2	10.5
	Std (10${}^{1}$)	2.27	1.88	2.01	1.48	3.95	3.68	5.43	5.56
	Rank	1	7	3.5	5.5	3.5	2	8	5.5
f8	Avg (10${}^{3}$)	4.96	9.83	6.83	3.16	8.31	7.46	1.06	8.45
	Std (10${}^{2}$)	7.68	11.6	18	4.02	26.5	14.6	26.1	30.2
	Rank	2	7	3	1	5	4	8	6
f9	Avg (10${}^{3}$)	5.77	8.55	7.40	8.71	6.76	7.38	5.53	6.13
	Std (10${}^{2}$)	7.62	2.72	4.87	3.71	8.38	6.92	6.69	7.20
	Rank	2	7	6	8	4	5	1	3

**Table 3:** Comparison results with variants of traditional optimization algorithms on hybrid functions.

Func		IGHHO	SCADE	CLPSO	BLPSO	IWOA	BMWOA	CDLOBA	RCBA
f10	Avg (10${}^{3}$)	1.23	4.33	2.76	2.16	4.01	1.62	1.34	1.34
	Std (10${}^{1}$)	4.73	87.8	55.8	32.1	175	16.4	7.77	9.84
	Rank	1	8	6	5	7	4	2.5	2.5
f11	Avg (10${}^{6}$)	1.15	2700	219	259	79.7	65.9	1.76	7.26
	Std (10${}^{6}$)	1.09	905	111	66.3	88.9	37.2	1.22	4.85
	Rank	1	8	6	7	5	4	2	3
f12	Avg (10${}^{4}$)	4.30	112,000	9000	5090	57	40.3	18	17.7
	Std (10${}^{4}$)	5.18	46,800	5030	2770	38.7	38.7	10.1	10.7
	Rank	1	8	7	6	5	4	3	2
f13	Avg (10${}^{4}$)	3.98	71.6	31	28.6	191	99.9	2.16	3.02
	Std (10${}^{4}$)	3.75	40.3	23.6	14.7	201	60.6	2.14	2.65
	Rank	3	6	5	4	8	7	1	2
f14	Avg (10${}^{3}$)	6.49	23,100	7550	6050	1580	92.3	90.1	70
	Std (10${}^{3}$)	8.69	20,200	6030	3360	4590	77.1	66.2	52.4
	Rank	1	8	7	6	5	4	3	2
f15	Avg (10${}^{3}$)	2.87	4.21	3.26	3.64	3.48	3.43	3.57	3.63
	Std (10${}^{2}$)	3.26	2.64	2.39	2.07	6.03	3.2	4.36	3.91
	Rank	1	8	2	7	4	3	5	6
f16	Avg (10${}^{3}$)	2.46	2.67	2.34	2.41	2.66	2.45	2.92	2.80
	Std (10${}^{2}$)	2.35	1.75	1.58	1.55	2.65	2.26	3.50	3.55
	Rank	4	6	1	2	5	3	8	7
f17	Avg (10${}^{5}$)	2.41	103	20.4	49.8	54.4	34.7	2.75	3.29
	Std (10${}^{5}$)	2.81	72.4	13.1	23.2	64.4	30.9	2.87	2.35
	Rank	1	8	4	6	7	5	2	3
f18	Avg (10${}^{3}$)	7.63	64,800	6120	9490	1160	749	323	357
	Std (10${}^{3}$)	5.22	28,800	4620	6460	2330	1170	83	257
	Rank	1	8	6	7	5	4	2	3
f19	Avg (10${}^{3}$)	2.73	2.89	2.58	2.69	2.80	2.66	2.96	2.99
	Std (10${}^{2}$)	2.22	1.37	1.53	1.26	1.86	2.39	2.18	2.30
	Rank	4	6	1	3	5	2	7	8

**Table 4:** Comparison results with variants of traditional optimization algorithms on composition functions.

Func		IGHHO	SCADE	CLPSO	BLPSO	IWOA	BMWOA	CDLOBA	RCBA
f20	Avg (10${}^{3}$)	2.49	2.60	2.52	2.54	2.58	2.55	2.6	2.61
	Std (10${}^{1}$)	6.31	3.19	3.8	1.47	4.92	5.46	6.13	6.5
	Rank	1	6.5	2	3	5	4	6.5	8
f21	Avg (10${}^{3}$)	5.14	5.8	4.71	2.79	7.28	4.8	7.10	7.24
	Std (10${}^{3}$)	2.42	7.85	1.82	0.0616	1.99	2.99	1.22	1.47
	Rank	4	5	2	1	8	3	6	7
f22	Avg (10${}^{3}$)	2.88	3.07	2.92	2.93	3.04	2.96	3.19	3.42
	Std (10${}^{1}$)	7.13	4.44	3.00	2.25	9.73	8.17	13.4	19.4
	Rank	1	6	2	3	5	4	7	8
f23	Avg (10${}^{3}$)	3.04	3.22	3.11	3.09	3.21	3.09	3.33	3.50
	Std (10${}^{1}$)	6.96	4.14	2.60	1.87	10.5	8.02	10.4	13.9
	Rank	1	6	4	2.5	5	2.5	7	8
f24	Avg (10${}^{3}$)	2.9	3.72	3.1	3.12	3.05	3.03	2.93	2.9
	Std (10${}^{1}$)	1.37	23.6	3.89	4.88	4.52	3.95	2.89	2.13
	Rank	1	8	6	7	5	4	3	2
f25	Avg (10${}^{3}$)	5.97	7.99	6.13	6.25	7.58	6.67	10	9.13
	Std (10${}^{3}$)	1.7	0.465	0.588	0.762	1.07	1.24	2.11	2.17
	Rank	1	6	2	3	5	4	8	7
f26	Avg (10${}^{3}$)	3.30	3.54	3.35	3.39	3.38	3.31	3.50	3.45
	Std (10${}^{1}$)	4.99	6.64	2.92	2.21	7.90	4.77	19.2	12.3
	Rank	1	8	3	5	4	2	7	6
f27	Avg (10${}^{3}$)	3.24	4.89	3.78	3.55	3.44	3.41	3.38	3.24
	Std (10${}^{1}$)	2.28	39.4	11.8	5.28	7.89	4.47	65.5	5.49
	Rank	1	8	7	6	5	4	3	2
f28	Avg (10${}^{3}$)	4.31	5.43	4.47	4.6	4.88	4.73	5.23	5.25
	Std (10${}^{2}$)	3.03	3.15	2.34	1.41	4.62	3.3	5.49	4.95
	Rank	1	8	2	3	5	4	6	7
f29	Avg (10${}^{5}$)	1.09	1710	94.9	117	59.6	55.8	8.7	25.2
	Std (10${}^{5}$)	1.81	576	56.6	44.4	51.3	37	6.92	21.2
	Rank	1	8	6	7	5	4	2	3

**Table 5:** Results of Wilcoxon signed-rank tests.

Wilcoxon Signed-Rank Test		
	***p*** **-Value (10${}^{-6}$)**	**n/w/t/l**
IGHHO vs. SCADE	3	29/29/0/0
IGHHO vs. CLPSO	114	29/24/0/5
IGHHO vs. BLPSO	292	29/24/0/5
IGHHO vs. IWOA	3	29/29/0/0
IGHHO vs. BMWOA	31	29/26/0/3
IGHHO vs. CDLOBA	73	29/27/0/2
IGHHO vs. RCBA	29	29/27/0/2

**Table 6:** Dataset.

Dataset	Features	Instance	Class
Wine	13	178	3
Zoo	16	101	7
Waveform noise	40	5000	3
Lung	57	27	3
Sonar	60	208	2
Hill_Valley	101	606	2
Clean1	168	476	2
Madelon	500	2600	2
Isolet	617	1559	26
CNAE	857	1080	9
Colon	2000	62	2
Leukemia	7129	72	2
Arcene	10,000	200	2

**Table 7:** Comparison of classification accuracy of IGHHO and other methods.

Dataset	IGHHO	HHO	EPO	SSA	SMA	BOA	WOA	ALO
Wine/Rank	96.00	95.14	96.57	96.14	93.86	93.29	95.14	95.71
	3	5	1	2	7	8	6	4
Zoo/Rank	94.75	91.50	94.75	94.25	92.50	90.50	92.50	93.25
	1	7	2	3	5.5	8	5.5	4
Waveform_noise/Rank	83.45	82.35	81.09	82.26	79.96	79.47	82.83	82.75
	1	4	6	5	7	8	2	3
Lung/Rank	96.00	81.00	91.00	79.00	79.00	77.00	85.00	83.00
	1	5	2	6	7	8	3	4
Sonar/Rank	92.32	89.02	92.20	90.85	88.17	87.68	90.12	90.49
	1	6	2	3	7	8	5	4
Hill_Valley/Rank	61.45	58.97	62.02	58.72	58.18	57.56	59.79	59.21
	2	5	1	6	7	8	3	4
Clean1/Rank	96.53	93.11	94.16	92.74	96.63	90.74	92.47	93.53
	2	5	3	6	1	8	7	4
Madelon/Rank	80.54	77.77	84.08	78.24	75.63	73.81	77.37	79.34
	2	5	1	4	7	8	6	3
Isolet/Rank	84.50	83.23	83.25	83.83	82.57	82.38	83.42	84.39
	1	6	5	3	7	8	4	2
CNAE/Rank	86.83	86.39	77.96	82.69	77.89	77.55	87.59	88.40
	3	4	6	5	7	8	2	1
Colon/Rank	96.25	90.00	90.42	85.83	96.25	86.67	91.67	89.58
	1.5	5	4	8	1.5	7	3	6
Leukemia/Rank	100.00	96.43	99.64	97.86	99.64	95.36	97.50	98.21
	1	7	2.5	5	2.5	8	6	4
Arcene/Rank	93.88	91.00	92.63	87.75	92.38	87.38	90.00	90.13
	1	4	2	7	3	8	6	5
Count	20.5	68	37.5	63	69.5	103	58.5	48
AvgRank	1.58	5.23	2.88	4.85	5.35	7.92	4.50	3.69
TotalRank	1	6	2	5	7	8	4	3

**Table 8:** Average computational time of IGHHO and other algorithms on FS problems.

Dataset	IGHHO	HHO	EPO	SSA	SMA	BOA	WOA	ALO
Wine/Rank	3.02	4.21	2.64	2.76	0.29	2.76	2.62	2.79
	7	8	3	5	1	4	2	6
Zoo/Rank	2.95	4.16	2.72	2.72	0.37	2.73	2.59	2.76
	7	8	3	4	1	5	2	6
Waveform_noise/Rank	15.60	30.68	10.59	15.07	2.35	12.81	20.15	22.28
	5	8	2	4	1	3	6	7
Lung/Rank	2.70	3.74	2.44	2.37	0.60	2.38	2.25	2.66
	7	8	5	3	1	4	2	6
Sonar/Rank	2.78	4.13	2.57	2.59	0.70	2.59	2.48	2.86
	6	8	3	5	1	4	2	7
Hill_Valley/Rank	2.99	4.41	2.78	3.01	0.39	2.95	2.76	3.35
	5	8	3	6	1	4	2	7
Clean1/Rank	2.98	4.85	2.72	3.09	1.06	3.01	2.99	3.98
	3	8	2	6	1	5	4	7
Madelon/Rank	36.30	88.45	13.26	48.84	7.47	40.69	52.57	64.98
	3	8	2	5	1	4	6	7
Isolet/Rank	18.51	38.02	8.72	24.62	6.25	21.48	27.71	30.02
	3	8	2	5	1	4	6	7
CNAE/Rank	20.85	46.98	10.20	17.94	7.09	15.45	27.86	33.87
	5	8	2	4	1	3	6	7
Colon/Rank	2.67	5.77	3.63	3.71	1.94	3.62	3.28	14.16
	2	7	5	6	1	4	3	8
Leukemia/Rank	3.16	9.09	5.20	6.00	4.63	5.76	5.35	44.62
	1	7	3	6	2	5	4	8
Arcene/Rank	5.12	16.83	7.99	16.49	6.28	14.69	10.68	66.85
	1	7	3	6	2	5	4	8
Count	55	101	38	65	15	54	49	91
AvgRank	4.23	7.77	2.92	5.00	1.15	4.15	3.77	7.00
TotalRank	5	8	2	6	1	4	3	7

## Data Availability

The data that support the findings of this study are available from UCI Machine Learning Repository. Restrictions apply to the availability of these data, which were used under license for this study.
